# Tuning the Hydrogen Evolution Activity of Co_2_NiO_4_ via Precursor-Controlled Synthesis

**DOI:** 10.3390/ijms27031584

**Published:** 2026-02-05

**Authors:** Abu Talha Aqueel Ahmed, Momin M. Mujtaba, Kafeel Ahmed Tufail Ahmed, Abu Saad Ansari, Sangeun Cho, Youngmin Lee, Sejoon Lee, Sankar Sekar

**Affiliations:** 1 Division of System Semiconductor, Dongguk University, Seoul 04620, Republic of Korea; abutalha.aa@dongguk.edu (A.T.A.A.); ymlee@dongguk.edu (Y.L.); sejoon@dongguk.edu (S.L.); 2 Department of Physics, M.S.G. Arts, Science & Commerce College, Malegaon-Camp, Malegaon 423203, India; mohammedmujtaba1318@gmail.com (M.M.M.); kafeelansari338@gmail.com (K.A.T.A.); 3 Nano Center Indonesia Research Institute, Puspiptek Street, South Tangerang 15314, Banten, Indonesia; saad@nano.or.id; 4 Quantum-Functional Semiconductor Research Center, Dongguk University, Seoul 04620, Republic of Korea

**Keywords:** electrocatalysts, hydrogen evolution reaction, Co_2_NiO_4_, turnover frequency, morphology variation

## Abstract

The realization of efficient and durable earth-abundant electrocatalysts for alkaline hydrogen evolution reaction (HER) is critical for scalable hydrogen production, yet remains limited by insufficient intrinsic activity. Herein, we demonstrate a precursor-controlled hydrothermal strategy that enables precise morphology and surface-state regulation of spinel Co_2_NiO_4_ directly grown on nickel foam, allowing a clear correlation between catalyst architecture and HER performance. By replacing urea with hexamethylenetetramine, an ultrathin, highly interconnected two-dimensional nanosheet network (CNO-HT) is obtained, which promotes efficient electron transport, rapid electrolyte penetration, and maximized exposure of catalytically active sites. Structural and spectroscopic analyses confirm the formation of phase-pure cubic Co_2_NiO_4_ with enriched mixed-valence Ni and Co species, favoring enhanced redox activity. The CNO-HT catalyst exhibits a low overpotential (86 mV at 10 mA cm^−2^) and a smaller Tafel slope (103 mV dec^−1^), significantly outperforming the urea-derived counterpart. Importantly, the catalyst maintains stable HER operation for 96 h at both 10 and 100 mA cm^−2^, with post-stability electrochemical analyses confirming preserved kinetics and interfacial properties. This work establishes precursor-regulated nanosheet engineering as general and scalable strategy to unlock the intrinsic catalytic potential of spinel metal oxides, offering actionable design principles for next-generation non-noble electrocatalysts for alkaline hydrogen production.

## 1. Introduction

The increasing global demand for energy, coupled with the urgent need to reduce carbon emissions, has intensified interest in sustainable and low-carbon energy carriers [1,2,3]. At present, the majority of hydrogen (H_2_) production relies on conventional routes such as steam methane reforming, coal gasification, and partial oxidation of hydrocarbons, which are energy-intensive and inevitably associated with significant CO_2_ emissions [4,5,6,7]. These processes undermine the environmental benefits of hydrogen and limit its role as a truly green fuel [8,9,10]. In contrast, electrochemical water electrolysis offers a clean and environmentally benign pathway for hydrogen generation, particularly when powered by renewable electricity [11]. This approach enables high-purity hydrogen production with zero direct carbon emissions, making it one of the most promising technologies for establishing a sustainable hydrogen economy. To realize efficient hydrogen generation via water electrolysis, the development of effective electrocatalysts for the hydrogen evolution reaction (HER) is essential [12,13,14,15]. While noble-metal catalysts such as platinum (Pt), Pt/C, and/or Pt-based catalysts exhibit outstanding HER activity, their scarcity and high cost significantly restrict large-scale deployment [9,16,17]. Therefore, non-precious transition-metal oxides (TMOs) have emerged as attractive alternatives to Pt-based catalysts owing to their earth abundance, chemical stability, and rich redox chemistry [9].

Among various TMOs, cobalt- and nickel-based oxides, particularly spinel-type Co_2_NiO_4,_ have received considerable attention for alkaline HER owing to their multiple accessible oxidation states and structural robustness [18,19,20,21]. For example, Wei et al. reported NiCo_2_O_4_ nanowire arrays synthesized via hydrothermal routes followed by annealing in air and Ar atmospheres, demonstrating HER overpotentials ranging from 226 to 104 mV at 10 mA cm^−2^ in alkaline media, depending on the annealing environment [22]. Similarly, He et al. prepared KCl salt-assisted NiCo_2_O_4_ nanoparticles through chemical-assisted synthesis followed by air annealing of the obtained black solid powder and demonstrated improved HER kinetics, albeit with limited HER durability (24 hours (h)) and endurance at 10 mA cm^−2^ [23]. Cheng et al. synthesized urchin-like Pt-NiCo_2_O_4_ through sonication and centrifugation process, and the catalyst electrode was fabricated using carbon black and PTFE solution, which exhibits the reduced overpotential of 40 mV at 10 mA cm^−2^ compared to pure NiCo_2_O_4_ (189 mV) [24]. However, the use of binders in catalyst electrode fabrication for powder samples can further deteriorate catalytic activity by creating inactive regions that hinder efficient ion and electron transport within bulk powder-based electrodes. Despite these advances, oxide-based catalysts alone generally suffer from intrinsically low electrical conductivity and suboptimal hydrogen adsorption kinetics, leading to sluggish charge-transfer kinetics and inferior intrinsic activity compared to noble metals, especially under practical alkaline conditions [25,26]. Overcoming these intrinsic limitations through rational material design remains a key challenge for advancing oxide-based HER catalysts. One effective strategy to enhance the intrinsic activity of metal oxides is morphology engineering, which directly influences surface area, active site exposure, and charge-transport pathways [27,28,29]. In particular, two-dimensional (2D) nanosheet architectures have emerged as highly promising platforms for electrocatalysis [29]. Compared to bulk counterparts and a thicker nanostructure, 2D nanosheets provide a large fraction of exposed surface atoms, shortened electron/ion diffusion lengths, and abundant edge sites that can serve as catalytically active centers [30,31]. Moreover, an ultrathin interconnected nanosheet can facilitate more efficient electrolyte penetration and improved electrical contact with conductive substrates, thereby accelerating HER kinetics [9].

Motivated by these advantages, this work focuses on the precursor-controlled hydrothermal strategy to construct two-dimensional Co_2_NiO_4_ nanosheet architectures on Ni foam (NF) and systematically evaluate their hydrogen evolution activity in alkaline media. By regulating the nature of the precursor chemistry, the growth kinetics and lateral extension of ultrathin nanosheets can be precisely tailored, enabling enhanced surface exposure and optimized electronic transport pathways. The resulting 2D Co_2_NiO_4_ interconnected nanosheets offer a high density of accessible active sites, shortened diffusion distances, and improved electrode-electrolyte interaction, which collectively promote faster HER kinetics despite the intrinsic conductivity limitations of metal oxides. The optimized CNO-HT catalyst delivers a markedly reduced overpotential of 86 mV at a cathodic current density of 10 mA cm^−2^, accompanied by a smaller Tafel slope of 103 mV dec^−1^, indicating the accelerated hydrogen evolution kinetics. In comparison, the CNO-U catalyst exhibits a relatively higher overpotential of 109 mV at the same driving current density, highlighting the critical role of precursor chemistry in governing catalytic activity. Across a wide range of applied current densities, CNO-HT catalyst consistently maintains a lower potential response than CNO-HMT catalyst, demonstrating superior catalytic robustness. Notably, CNO-HT also shows outstanding chronopotentiometric stability, sustaining stable operation at a current density of 10 mA cm^−2^ and even at 100 mA cm^−2^ over prolonged duration of 96 h. The electrochemically active surface area (*ECSA*), Nyquist impedance, and Turnover frequency (*TOF*) analyses further confirm that CNO-HT catalyst possesses higher site utilization efficiency and enhanced intrinsic reaction kinetics relative to CNO-U catalyst film. These performance gains are attributed to the well-developed two-dimensional nanosheet architecture, intimate electrical contact with the Ni foam substrate, and a continuous conductive network, which together facilitate efficient electron/ion transport and promote rapid hydrogen evolution in an alkaline KOH medium. This study establishes a clear morphological structure-activity correlation between precursor selection, nanosheet formation, and HER performance, providing valuable design principles for advancing oxide-based electrocatalysts toward efficient hydrogen generation.

## 2. Results and Discussion

### 2.1. Morphological and Compositional Properties of CNO-U and CNO-HT Electrode Films

**Figure 1:**
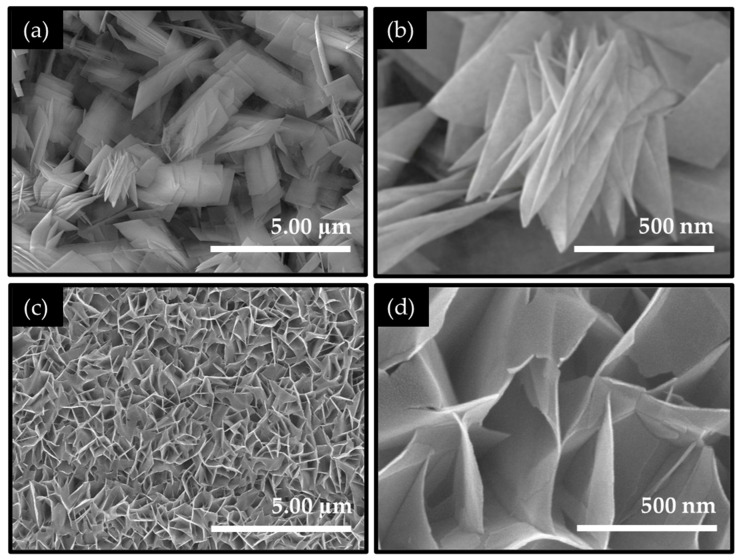
FESEM images of Co_2_NiO_4_ electrode films synthesized using different precursors. Low- and high-magnification images of (**a**,**b**) CNO-U and (**c**,**d**) CNO-HT electrode films.

The surface morphology and microstructural evolution of the synthesized Co_2_NiO_4_ electrodes were investigated by field-emission scanning electron microscopy (FESEM), as shown in Figure 1. The distinct differences in nanosheet thickness, alignment, and interconnectivity are observed between the CNO-U and CNO-HT electrode films, highlighting the decisive role of precursor chemistry in directing nanostructure formation. Figure 1a,b present the low- and high-magnification FESEM images of the CNO-U electrode film. The surface is composed of thick plate-like nanosheets that are randomly oriented and loosely stacked. These nanosheets exhibit a broad lateral dimension, resulting in partial overlap and agglomeration. The observed morphology is indicative of a growth mechanism governed by gradual nucleation and relatively slow anisotropic crystal growth, in which urea serves as a gentle and leisurely source of hydroxide ions (OH^−^). The controlled hydrolysis of urea promotes the progressive formation of metal hydroxide intermediates, enabling sustained lattice expansion along multiple crystallographic directions and ultimately leading to the development of thicker nanosheet structures. In contrast, the CNO-HT electrode displays a markedly different morphology, as shown in Figure 1c,d. The NF surface is uniformly covered by a dense, well-aligned two-dimensional nanosheet network, where ultrathin nanosheets are vertically and laterally interconnected, forming a highly porous architecture. The nanosheets for the CNO-HT electrode are significantly thinner and more sharply defined, with abundant open channels between adjacent sheets [9,32]. This interconnected 2D framework provides extensive surface exposure and facilitates efficient electrolyte penetration.

The pronounced morphological refinement observed in CNO-HT can be attributed to the distinct role of HT during the hydrothermal growth. It decomposes more rapidly under hydrothermal conditions, generating OH^−^ ions at a higher and more uniform rate. This accelerated alkalization promotes rapid nucleation over growth, suppressing excessive crystal thickening while favoring lateral expansion of nanosheets. In addition, the coordination ability of HT with metal cations can transiently regulate local ion concentrations, directing the preferential two-dimensional growth and preventing nanosheet restacking. As a result, CNO-HT evolves into a highly ordered nanosheet ensemble with intimate intersheet contact. Nonetheless, the FESEM analysis clearly demonstrates that precursor selection critically governs the nanosheet growth mechanism, transitioning from comparatively thick, randomly stacked nanosheets in CNO-U to a thin, aligned, and interconnected 2D nanosheet network in CNO-HT. This structural transformation is expected to significantly enhance active-site accessibility, shorten ion/electron transport pathways, and improve electrode-electrolyte interaction, thereby contributing to the superior hydrogen evolution performance of the CNO-HT electrode film. Thereafter, the energy-dispersive X-ray spectroscopy (EDAX, Figure S1a,b) was employed to examine the elemental composition of the CNO-U and CNO-HT electrode films. The spectra confirm the exclusive presence of cobalt, nickel, and oxygen in both electrode films, with no detectable impurity elements, indicating high compositional purity. The relative elemental distribution in both electrodes is consistent with the expected stoichiometry of the Co_2_NiO_4_ spinel phase, demonstrating effective incorporation of Co and Ni cations within the oxide lattice. A slight variation in the relative Co and Ni signal intensities is observed between CNO-U and CNO-HT, suggesting that the precursor chemistry influences local cation distribution during growth. This elusive compositional modulation, when combined with nanosheet thinning and enhanced interconnectivity in the CNO-HT electrode film, might contribute to differences in electronic structure and catalytic site availability. The comprehensive EDAX results corroborate the successful synthesis of near-stoichiometric Co_2_NiO_4_ in both electrode films.

### 2.2. Crystallographic Properties of CNO-U and CNO-H Electrode Films

**Figure 2:**
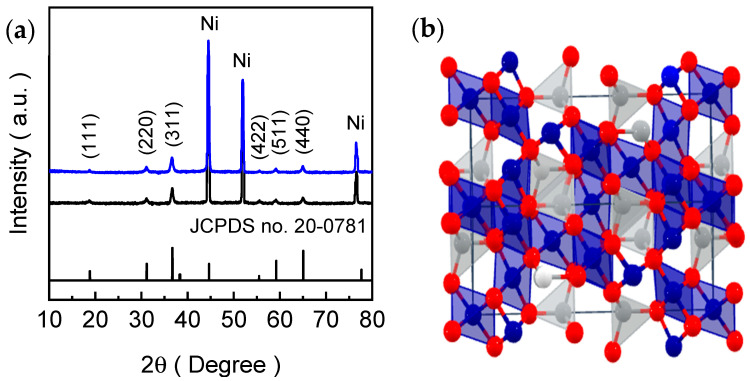
(**a**) XRD patterns of CNO-U (black) and CNO-HT (blue) electrode films grown on nickel foam, along with the relevant JCPDS card for Co_2_NiO_4_ (JCPDS No. 20-0781). The marked reflections correspond to the characteristic crystallographic planes of the spinel structure, while the additional peaks originate from the Ni foam substrate. (**b**) Schematic illustration of the cubic spinel Co_2_NiO_4_ crystal structure, showing the distribution of Co/Ni (blue and gray spheres) cations within tetrahedral and octahedral coordination environments in the oxygen (red sphere) framework.

The crystallographic structure and phase purity of the CNO-U and CNO-HT electrode films were investigated by X-ray diffraction (XRD), as shown in Figure 2a. Both electrode films display characteristic diffraction peaks at approximately 18.73°, 31.09°, 36.61°, 55.58°, 59.16°, and 64.99°, which are indexed to the (111), (220), (311), (422), (511), and (440) planes of the cubic spinel Co_2_NiO_4_ phase, respectively, in close agreement with the standard reference JCPDS card No. 20-0781 [33,34,35,36]. Among these reflections, the relatively intense (311) peak indicates a preferred orientation typical of spinel Ni-Co oxides. In addition, the peaks located at ~44.54°, 51.96°, and 76.50° originate from the metallic Ni foam substrate and correspond to the (111), (200), and (220) planes of fcc Ni, confirming the direct growth of Co_2_NiO_4_ on the conductive NF support. Importantly, no additional reflections associated with impurity phases (e.g., NiO or Co_3_O_4_) are detected, confirming the formation of single-phase spinel Co_2_NiO_4_ in both CNO-U and CNO-HT. The spinel crystallographic arrangement is illustrated schematically in Figure 2b, where the oxygen sublattice forms a close-packed framework and the metal cations occupy tetrahedral and octahedral interstitial sites, yielding the characteristic B_2_AO_4_-type spinel structure. This lattice architecture enables mixed-valence redox flexibility and provides multiple metal-oxygen coordination environments that are relevant to electrochemical reactions.

The lattice constant (*a* = 5.71 Å) of the cubic spinel Co_2_NiO_4_ was estimated from the indexed XRD peaks, and the corresponding unit-cell volume (*V* = *a*^3^ = 186.169 Å^3^) was determined, confirming the formation of a well-defined spinel structure. Notably, although both electrodes share the same spinel phase, the CNO-HT exhibits slightly broader and less intense peaks compared with CNO-U, suggesting a reduced coherent crystallite size and/or increased microstrain. From the (311) reflection, the analysis yields crystallite sizes of ~18.9 and 15.7 nm for the CNO-U and CNO-HT, respectively. To further separate size and strain contributions, Williamson–Hall analysis using the (220), (311), (511) and (440) reflections gives crystallite sizes of ~16.4 nm (CNO-U) and ~15.6 nm (CNO-HT), in reasonable agreement with the Scherrer estimates, and indicates low microstrain in both samples. This observation is consistent with the morphological results, where the nanosheet thickness of CNO-HT electrode is reduced relative to CNO-U electrode film. Thinner nanosheets generally correspond to smaller coherent diffraction domains along the thickness direction, which can manifest as peak broadening in XRD. This crystallite alteration and defect-associated lattice disorder can increase the density of coordinatively unsaturated surface sites and facilitate charge-transfer processes, offering a plausible structural basis for the superior HER activity of the CNO-HT electrode film.

### 2.3. Chemical Bonding States of CNO-H Electrode Film

**Figure 3:**
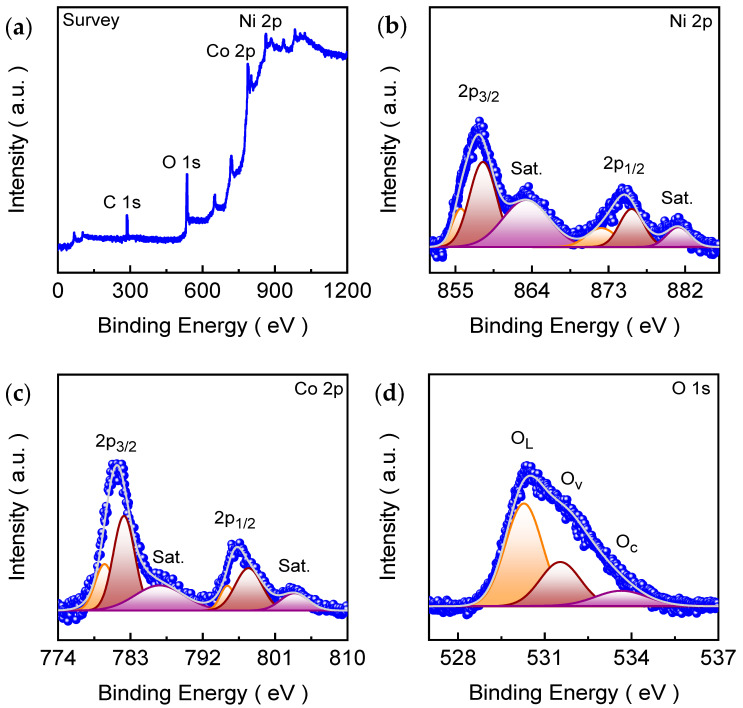
XPS analysis of the CNO-HT electrode: (**a**) survey spectrum confirming the presence of Co, Ni, and O elements. The high-resolution deconvoluted spectra of (**b**) Ni 2p, (**c**) Co 2p, and (**d**) O 1s emission states.

The X-ray photoelectron spectroscopy (XPS) was employed to investigate the surface elemental composition and chemical states of the CNO-HT electrode film. The wide-scan survey spectrum (Figure 3a) clearly reveals the presence of Co, Ni, and O as the dominant elements, confirming the successful formation of the targeted binary oxide. A weak C 1s signal is also observed at 284.13 eV, which is commonly attributed to adventitious carbon contamination arising from atmospheric exposure during sample handling and is typically used as a reference for binding energy calibration [37]. Importantly, no additional peaks associated with impurity elements are detected, indicating high surface purity and effective incorporation of cobalt and nickel within the oxide lattice. The clear identification of Co and Ni signals in the survey spectrum confirms the coexistence of both metal cations at the surface, which is essential for the mixed-valence redox chemistry characteristic of spinel Co-Ni oxides [38]. This compositional integrity provides a reliable basis for subsequent high-resolution analysis of the Co 2p, Ni 2p, and O 1s regions to elucidate the oxidation states, defect chemistry, and their correlation with hydrogen evolution activity. Figure 3b shows the high-resolution Ni 2p XPS spectrum of the electrode, which was deconvoluted using a Gaussian curve-fitting model to resolve the individual chemical-state contributions. The spectrum displays two dominant spin–orbit components centered in the regions of Ni 2p_3/2_ (857.83 eV) and Ni 2p_1/2_ (887.88 eV), accompanied by characteristic shake-up satellite (“Sat.” at 863.27 and 881.16 eV) features. These satellites originate from final-state effects and multiple splitting commonly associated with partially filled Ni 3d orbitals, and their pronounced intensity is generally considered an indicator of strong electronic interactions between Ni centers and the oxygen lattice [39]. The main Ni 2p_3/2_ peak at lower binding energy can be fitted into contributions associated with Ni^2+^ (855.64 eV) and Ni^3+^ (858.21 eV) species, indicating the coexistence of multiple Ni valence states at the surface of the Co_2_NiO_4_ lattice [40,41].

Similarly, the Ni 2p_1/2_ region at higher binding energy exhibits corresponding fitted components that mirror the Ni^2+^/Ni^3+^ contributions, consistent with spin–orbit coupling. The presence of distinct satellite peaks on the high-binding-energy side of both 2p_3/2_ and 2p_1/2_ further supports the assignment of mixed valence state of Ni in the coordination environment. This is typical of nickel-based spinel oxides, where nickel exists in mixed oxidation states and experiences strong ligand-metal charge transfer effects [42,43]. The high-resolution deconvoluted Co 2p spectrum is shown in Figure 3c to analyze the surface cobalt chemical states. The spectrum also exhibits two main spin–orbit components corresponding to Co 2p_3/2_ (781.33 eV) and Co 2p_1/2_ (796.29 eV), accompanied by characteristic shake-up satellite (“Sat.” at 786.61 and 803.40 eV) peaks. This spectral profile is typical of cobalt-based spinel oxides and confirms the presence of mixed cobalt valence states at the surface. The Co 2p_3/2_ peak can be fitted into contributions arising from Co^3+^ (779.79 eV) and Co^2+^ (782.19 eV) species, while the corresponding Co 2p_1/2_ region displays similar Co^3+^/Co^2+^ components situated at 795.04/797.68 eV, respectively, consistent with spin–orbit coupling of the degenerate states [44]. The appearance of pronounced satellite features adjacent to both main peaks supports the assignment of cobalt in an oxide-coordinated environment and indicates strong final-state effects associated with partially filled Co 3d orbitals. In general, comparatively intense satellites are commonly linked with Co^2+^ contributions, while Co^3+^ components tend to appear with reduced satellite intensity, suggesting that both oxidation states coexist and contribute to the overall electronic structure of Co_2_NiO_4_ [45,46]. The coexistence of Co^3+^/Co^2+^ redox centers is particularly beneficial for electrochemical functionality, as it enables flexible charge compensation and facilitates rapid electron exchange within the spinel lattice.

The surface oxygen chemistry of the CNO-HT electrode was further examined using high-resolution O 1s XPS (Figure 3d). The deconvoluted O 1s spectrum reveals three distinct components that represent different oxygen environments in the oxide lattice. The dominant low-binding-energy contribution corresponds to lattice oxygen (O_L_, M-O-M) within the spinel framework of Co_2_NiO_4_ [46,47]. This component confirms the formation of a well-developed metal-oxygen network consistent with the spinel structure identified by XRD. A second component at intermediate binding energy is commonly assigned to defect-related oxygen species (O_v_), including oxygen vacancies and oxygen associated with under-coordinated metal sites. The presence of this O_v_ contribution suggests that the CNO-HT surface contains a measurable fraction of defect sites and locally distorted coordination environments. These defect features are often beneficial for electrocatalysis because they can modify local electron density around Co/Ni centers and increase the density of unsaturated surface sites, thereby improving adsorption of reaction intermediates and facilitating charge transfer under alkaline HER conditions [48]. The third, high-binding-energy feature, labeled O_c_, is attributed to surface hydroxyl groups and/or adsorbed oxygen-containing species (e.g., –OH and H_2_O) that form naturally upon exposure to air or during alkaline electrolyte contact [49]. This surface component reflects the chemically active and hydrophilic nature of the oxide surface and is frequently observed for transition-metal oxides prepared under aqueous conditions. Nonetheless, the XPS results reveal that the CNO-HT electrode possesses a defect-rich spinel oxide surface characterized by robust metal-oxygen coordination, abundant oxygen-related defects, and coexisting Ni^2+^/Ni^3+^ and Co^3+^/Co^2+^ redox couples. This mixed-valence electronic structure enhances surface redox flexibility, promotes efficient interfacial charge transfer, and optimizes the adsorption/desorption energetics of reaction intermediates. This synergistic modulation of lattice oxygen, transition-metal valence states, and surface defects provides a strong electronic foundation for the accelerated catalytic kinetics and superior HER performance of the interconnected nanosheet architecture.

### 2.4. Electrochemical Properties of CNO-U and CNO-H Catalyst Films

**Figure 4:**
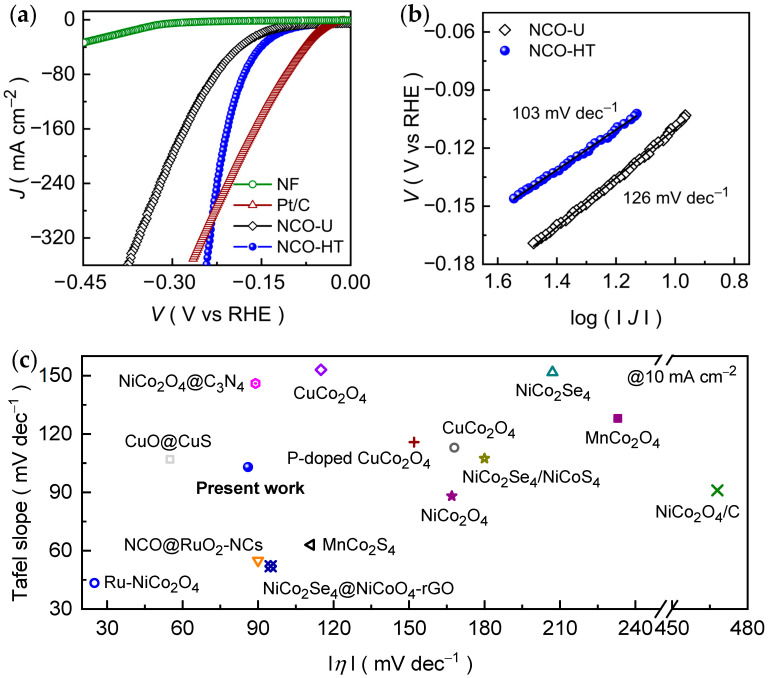
Electrochemical HER performances of CNO-U and CNO-HT nanosheet catalyst film examined in an alkaline KOH electrolyte (1 M) medium. (**a**) LSV curves and (**b**) Tafel slopes. (**c**) Comparative HER activity of the optimized CNO-HT and various previously reported TMOs-based catalysts. Notably, the detailed descriptions are presented in the Table S1.

The HER activity of the prepared CNO-U and CNO-HT catalyst films was investigated in 1.0 M KOH using linear sweep voltammetry (LSV) at a scan rate of 1 mV s^−1^. As shown in Figure 4a, bare NF exhibits negligible catalytic activity over the measured potential range, confirming that its contribution to hydrogen evolution is minimal under identical testing conditions. Interestingly, the CNO-HT interconnected nanosheet catalyst delivers the lowest overpotential of 86 mV to reach a cathodic current density of 10 mA cm^−2^, clearly outperforming the CNO-U nanosheets (109 mV). The reduced overpotential of CNO-HT indicates more favorable HER kinetics, which can be attributed to its optimized interconnected 2D nanosheet morphology and improved electrochemically accessible surface (Figure S2). Moreover, at an elevated current density of 25, 50, 75, 100, and 300 mA cm^−2^ the CNO-HT nanosheet catalyst consistently maintains lower overpotentials of 132, 161, 176, 189, and 235 mV compared to the CNO-U catalyst (161, 199, 223, 243, and 348 mV), demonstrating the superior catalytic performance across a wide operational range. Notably, the polarization curve of CNO-HT shows a more gradual increase in overpotential with increasing current density, reflecting improved charge-transfer efficiency and enhanced tolerance toward high-rate hydrogen evolution. Further, the performance difference between the two catalysts becomes more pronounced at higher currents, highlighting the structural advantage of the interconnected 2D nanosheet architecture under diverse operating conditions. For benchmarking purposes, the HER activity of a commercial Pt/C catalyst was evaluated under identical experimental conditions, and the obtained LSV curve for the Pt/C catalyst is included in Figure 4a. As expected, Pt/C exhibits superior HER activity at low overpotentials owing to its noble-metal nature. Notably, at higher current densities, the overpotential of the optimized CNO-HT catalyst becomes closely comparable to that of Pt/C, indicating competitive performance under increased driving conditions. This behavior highlights the potential of CNO-HT as an earth-abundant and durable alternative to noble-metal catalysts for alkaline HER, particularly for high-current-density operation.

To gain deeper insight into the HER kinetics of the prepared CNO-U and CNO-HT nanosheet catalysts, Tafel slope analysis was carried out based on the corresponding polarization curves (Figure 4a). Figure 4b presents the Tafel plots of the CNO-U and CNO-HT nanosheet catalyst films. The extracted Tafel slopes for CNO-U and CNO-HT catalysts are 126 mV dec^−1^ and 103 mV dec^−1^, respectively, clearly indicating the faster reaction kinetics for the CNO-HT nanosheet catalyst. Generally, in an alkaline electrolyte, the HER proceeds through a multistep mechanism involving the formation and subsequent removal of adsorbed hydrogen intermediates on the catalyst surface. The initial step is the Volmer reaction, in which water molecules are reduced to generate adsorbed hydrogen species (MH_ads_) and OH^−^, represented as:

H_2_O + M + e^−^ → OH^−^ + MH_ads_ (Volmer step, 120 mV dec^−1^), (1)

The generated MH_ads_ species are then converted into molecular hydrogen either through the electrochemical desorption process (Heyrovsky step):

H_2_O + MH_ads_ + e^−^ → M + OH^−^ + H_2_ (40 mV dec^−1^), (2)

or via the chemical recombination of two adsorbed hydrogen atoms (Tafel step):

2 MH_ads_ → 2 M + H_2_ (40 mV dec^−1^), (3)

The obtained Tafel slopes for both CNO-U (126 mV dec^−1^) and CNO-HT (103 mV dec^−1^) fall close to the theoretical value associated with the Volmer step, suggesting that water dissociation is the dominant rate-limiting process for HER on these oxide catalysts in alkaline media. Importantly, the lower Tafel slope of CNO-HT compared to CMO-U reflects a reduced kinetic barrier for hydrogen evolution, indicating more efficient charge transfer and accelerated reaction kinetics. This analysis is further supported by the *TOF* results (Figure S1c), which show that CNO-HT delivers a higher turnover frequency of 0.25 s^−1^ than CNO-U (0.077 s^−1^) at 230 mV, confirming that the improved HER performance originates from enhanced intrinsic site activity enabled by the interconnected nanosheet morphology. The enhanced HER activity of CNO-HT is mainly attributed to its well-defined interconnected two-dimensional, comparatively thinner nanosheet network, which provides increased exposure of active sites (Figure S2), improved electrolyte accessibility, and reduced ion-diffusion resistance. In addition, the direct growth of the catalyst on Ni foam ensures efficient electron transport and mechanical stability during the HER operation. Importantly, the HER performance metrics achieved by CNO-HT are comparable to those of many reported non-precious Ni- and Co-based metal oxide electrocatalysts in alkaline KOH electrolyte medium, as summarized in Figure 4c and Table S1. These results demonstrate that precursor-controlled synthesis is an effective and reliable (Figure S3) strategy for enhancing the intrinsic HER activity of Co_2_NiO_4._

**Figure 5:**
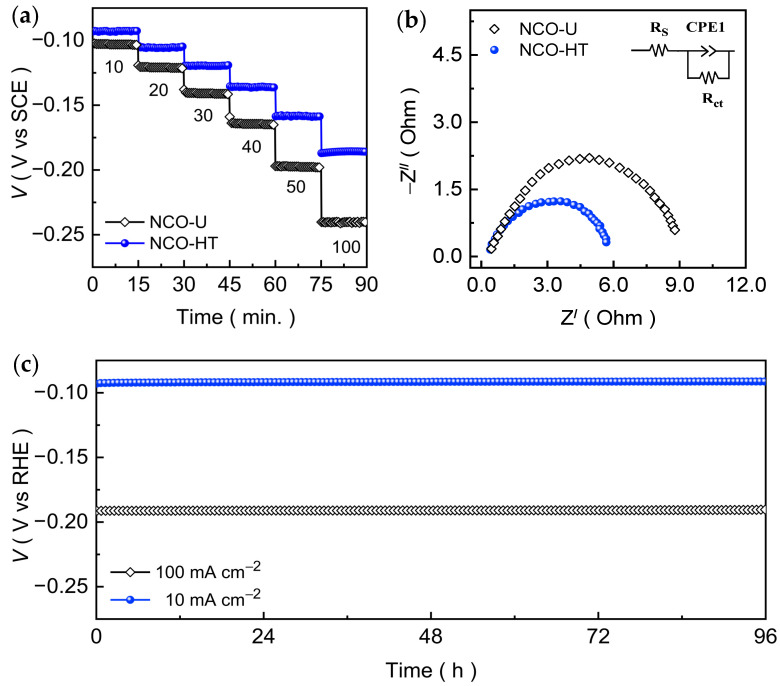
(**a**) Voltage step profile being function of current density and (**b**) Nyquist impedance plots for CNO-U and CNO-HT catalyst films evaluated in an alkaline KOH medium. (**c**) Chronopotentiometric stability curves recorded at 10 and 100 mA cm^−2^ examined for prolonged duration over 96 h.

Thereafter, the potential response of the CNO-U and CNO-HT nanosheet catalysts under increasing current demand, chronopotentiometric voltage-step measurements were conducted in 1.0 M KOH. Figure 5a displays the voltage-step profiles recorded by sequentially increasing the applied current density from 10 to 50 mA cm^−2^ in increments of 10 mA cm^−2^, followed by a further upsurge to 100 mA cm^−2^. Each current step was maintained for 15 min. to ensure stabilization of the catalyst potential before switching to the next current level. Both CNO-U and CNO-HT nanosheet catalysts exhibit well-defined and stable potential plateaus at each applied current density, indicating good electrochemical reversibility and steady-state operation during the measurement. Notably, the CNO-HT interconnected nanosheet catalyst consistently requires a lower overpotential than CNO-U catalyst across the entire current range, reflecting its superior charge-transfer characteristics and improved reaction kinetics [48]. As the current density increases, the potential rise remains gradual for CNO-U, whereas CNO-HT shows a comparatively steeper voltage increase, suggesting the higher polarization losses at elevated current densities. The absence of abrupt voltage fluctuations during both current escalation and holding periods confirms the robust electrode-electrolyte interface and strong mechanical integrity of the catalyst films. The relatively small potential hysteresis observed upon reaching higher current densities further indicates efficient mass transport and rapid electron conduction, particularly for the interconnected nanosheet network. These results demonstrate that the CNO-HT catalyst can sustain stable HER operation under dynamically varying current conditions, highlighting its suitability for practical hydrogen evolution at moderate-to-high current densities.

To gain deeper insight into the interfacial charge-transfer behavior during HER, electrochemical impedance spectroscopy (EIS) measurements were performed at the HER operating potential in 1.0 M KOH. Figure 5b presents the Nyquist plots of the CNO-U and CNO-HT nanosheet catalyst films. The impedance spectra consist of a quasi-semicircle, which is characteristic of the charge-transfer resistance (*R*_ct_) associated with the HER process at the electrode-electrolyte interface. Notably, the CNO-HT interconnected nanosheet catalyst exhibits a substantially smaller semicircle diameter compared to CNO-U, indicating a markedly lower *R*_ct_ (Table S2) and thus faster interfacial electron-transfer kinetics. This reduced charge-transfer resistance directly correlates with the superior HER activity observed in the polarization and Tafel analyses. The improved charge transport can be attributed to the thin, highly interconnected 2D nanosheet architecture of CNO-HT, as observed in FESEM (Figure 1), which provides shortened electron pathways and a larger number of exposed electrochemically active sites. Moreover, the intimate contact between the ultrathin nanosheets and the conductive Ni foam substrate in CNO-HT enhances electrical connectivity and minimizes contact resistance, further facilitating rapid electron injection into the active sites.

The long-term electrochemical durability of the optimized CNO-HT nanosheet catalyst was evaluated by chronopotentiometric measurements in alkaline KOH electrolyte, as shown in Figure 5c. The stability tests were carried out for 96 h at two representative cathodic current densities of 10 and 100 mA cm^−2^, corresponding to moderate and high hydrogen production regimes. At 10 mA cm^−2^, the catalyst exhibits a nearly constant potential throughout the entire test duration, indicating excellent operational stability with negligible degradation. Upon increasing the applied current density to 100 mA cm^−2^, CNO-HT again maintains a stable voltage response without noticeable potential drift, demonstrating robust performance even under high current rate. Further, the sustained stability at both current densities highlights the mechanical integrity of the catalyst layer and the preservation of efficient interfacial charge-transfer kinetics during prolonged HER operation. This behavior is closely associated with the thin, interconnected 2D nanosheet architecture, which ensures strong adhesion to the Ni foam substrate, facilitates rapid electron transport, and promotes efficient hydrogen bubble release, thereby preventing catalyst detachment or active-site blockage. Post-stability XRD (Figure S4a) analysis confirms that the spinel Co_2_NiO_4_ phase remains intact after prolonged HER operation, demonstrating excellent structural robustness of the electrode. Nonetheless, the excellent chronopotentiometric stability is fully consistent with the post-stability LSV (Figure S4b) and EIS (Figure S4c) measurements, which show nearly unchanged polarization behavior and charge-transfer resistance after long-term testing. These results collectively confirm the outstanding durability and electrochemical robustness of the CNO-HT catalyst, underscoring its suitability for sustained alkaline hydrogen evolution at both low and high current densities.

## 3. Materials and Methods

### 3.1. Materials

Nickel nitrate hexahydrate (Ni(NO_3_)_2_·6H_2_O, ≥99%), potassium hydroxide (KOH, ≥85%), cobalt nitrate hexahydrate (Co(NO_3_)_2_·6H_2_O, ≥99%), urea (U, CO(NH_2_)_2_, ≥99%), hexamethylenetetramine (HT, C_6_H_12_N_4_, ≥99%), ammonium fluoride (NH_4_F, ≥98%), ethanol (CH_3_CH_2_OH, ≥99.5%), acetone (CH_3_COCH_3_, ≥99.5%), and hydrochloric acid (HCl, 37%) were purchased from standard commercial supplier (Sigma-Aldrich, St. Louis, MO, USA) and used as received without further purification. Deionized (DI) water was used throughout all experiments. Commercial three-dimensional porous nickel foam (NF, thickness ~ 1.6 mm, Alantum, Seoul, Republic of Korea) served as the conductive substrate, and it was cleaned prior to use. The NF substrates were cut into desired dimensions (size = 5 × 1 cm^2^) and sequentially cleaned by ultrasonication in CH_3_COCH_3_, CH_3_CH_2_OH, and deionized (DI) water (10 min each). To remove surface oxides, the foam was briefly treated in dilute HCl solution, followed by thorough rinsing with DI water and CH_3_CH_2_OH followed by drying in vacuum ambient.

### 3.2. Synthesis of CNO-U and CNO-HT Catalyst Film

**Figure 6:**
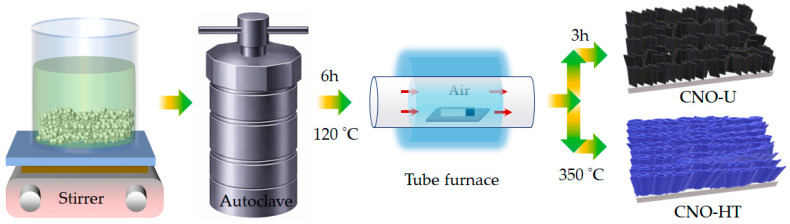
Schematic illustration of the precursor-controlled synthesis of Co_2_NiO_2_ catalyst films. The metal precursor solution is prepared under continuous stirring, followed by hydrothermal growth in a sealed autoclave. The obtained precursor films are subsequently annealed in air using a tube furnace, leading to the formation of Co_2_NiO_4_ with distinct nanosheet morphologies depending on the precursor chemistry, yielding CNO-U and CNO-HT structures.

The preparation procedures of the Co_2_NiO_4_ nanostructure films were synthesized on Ni foam through a facile hydrothermal technique described as follows. In a typical procedure, a stoichiometric amount of Ni^2+^ (Ni(NO_3_)_2_·6H_2_O) and Co^2+^ (Co(NO_3_)_2_·6H_2_O) salts were dissolved in DI water to form a clear metal precursor solution with a Ni:Co molar ratio of 1:2. The CO(NH_2_)_2_ (36 mmol) and NH_4_F (12 mmol) was then added in an aqueous mixture that was prepared in a glass beaker (100 mL) under continuous stirring at room temperature. The cleaned NF substrate was immersed in the formed solution, transferred to a Teflon-lined autoclave, and heated at 120 °C for 6 h. After cooling to room temperature, the obtained precursor films were removed, thoroughly rinsed with DI water and ethanol, and dried overnight, followed by air annealing at 350 °C for 3 h to obtain the desired Co_2_NiO_4_ (CNO-U) catalyst film (Figure 6). For comparison, the CNO-HT catalyst film was synthesized under identical hydrothermal conditions, with hexamethylenetetramine used in place of urea as the base source in the precursor solution. The overall precursor-controlled synthesis and post-annealing process for the Co_2_NiO_4_ electrode films is schematically illustrated in Figure 6.

### 3.3. Material Characterization

The crystallographic structures and phase purity of the synthesized Co_2_NiO_4_ nanostructures were systematically examined using XRD (Rigaku Smartlab, Akishima, Japan) employing Cu Kα radiation with a wavelength of 1.5406 Å. The diffraction patterns were recorded in the 2θ range of 20° to 80° at room temperature to identify the characteristic lattice planes and confirm phase formation. The morphological features and surface topography of the Co_2_NiO_4_ nanostructures were analyzed by FESEM (JSM-6701F, JEOL, Tokyo, Japan), providing detailed insights into particle size, shape, and surface texture. In addition, the elemental composition and spatial distribution of the constituent elements were determined through EDAX integrated with the FESEM system, enabling qualitative and semi-quantitative elemental mapping across the electrode surface. The surface chemical states and elemental composition were systematically investigated using the XPS conducted on a PHI 5000 VersaProbe Scanning Microprobe (ULVAC-PHI, PHI 5000 VersaProbe, Chigasaki, Japan). The high-resolution core-level spectra were collected for all relevant elements, and the binding energy positions were calibrated using the adventitious carbon C 1s peak centered at 286.85 eV to correct for any surface charging effects.

### 3.4. Catalytic HER Test of CNO-U and CNO-HT Catalysts

The hydrogen evolution reaction performances of the CNO-U and CNO-HT catalysts were systematically evaluated using a VersaSTAT electrochemical workstation (Ametek Scientific Instruments, Berwyn, PA, USA) in a conventional three-electrode configuration. All electrochemical measurements were conducted in 1.0 M KOH aqueous electrolyte at ambient temperature. The as-prepared catalyst films grown on nickel foam were directly employed as working electrodes, whereas a saturated calomel electrode (SCE) and a graphite rod served as the reference and counter electrodes, respectively. Polarization behavior was examined by linear sweep voltammetry (LSV) conducted within the potential range of 0.0 to −1.5 V (vs. SCE) at a scan rate of 1.0 mV s^−1^. The experimentally obtained potentials referenced to SCE were converted to the reversible hydrogen electrode (RHE) scale using the following relationship:

*E*_RHE_ = *E*_SCE_ + *E*_SCE_^∘^ + (0.059 × *pH*) (4)

To account for solution and contact resistance, *iR* (*JR*s) compensation was applied, where the solution resistance (*R*_S_) was determined from the high-frequency intercept of the Nyquist plot obtained by electrochemical impedance spectroscopy (EIS). The HER overpotential (*η*) was then calculated using the following equation:

*η* = *E*_RHE_ − (*J* × *R*s), (5)

The reaction kinetics were evaluated by constructing the Tafel plots from the steady-state regions of the polarization curves. The resulting data were fitted to the classical logarithmic relationship between overpotential and current density to extract the kinetic parameters, where the slope reflects the intrinsic reaction rate of the electrode surface:

*η* = *a* + (*b* × log(*J*)), (6)

where *b* is the Tafel slope and *a* is the arbitrary constant of the above equation. The electrochemical robustness of the CNO-U and CNO-HT catalysts was further examined via constant-current operation over prolonged time intervals to monitor potential fluctuations under continuous hydrogen generation. To quantify the number of electrochemically accessible active sites, the electrochemically active surface area (*ECSA*) was estimated using capacitive current analysis. This was achieved by recording cyclic voltammograms in a narrow potential window free of Faradaic processes at multiple scan rates, from which the non-Faradaic capacitive current density (*J*_NFC_) contribution was determined using the following equations:

*ECSA* = *C*_NFC_/*C*_e_, (7)

*C*_NFC_ = *J*_NFC_/*v*, (8)

where *C*_e_, and *v* are the KOH electrolyte capacitance and scan rate, respectively [50]. Further, the charge-transfer behavior at the electrode-electrolyte interface was investigated using electrochemical impedance spectroscopy. Measurements were carried out under HER-relevant bias conditions by applying a small sinusoidal amplitude (10 mV), while sweeping the frequency from high to low values (0.01–10 kHz). The resulting impedance spectra were analyzed to determine the charge-transfer resistance and assess interfacial electron-transport efficiency.

## 4. Conclusions

In summary, we have demonstrated a precursor-controlled synthesis strategy to rationally tailor the morphology, electronic structure, and electrocatalytic performance of spinel Co_2_NiO_4_ toward alkaline hydrogen evolution. By simply regulating the hydroxyl-releasing precursor during hydrothermal growth, distinct nanosheet architectures were obtained, enabling a direct structure-performance correlation. Compared with the CNO-U, the CNO-HT catalyst develops an ultrathin, highly interconnected two-dimensional nanosheet network with enhanced structural uniformity and intimate contact with the conductive Ni foam substrate. Comprehensive structural and surface analyses confirm the formation of a phase-pure cubic spinel Co_2_NiO_4_ framework with enriched mixed-valence Ni^2+^/Ni^3+^ and Co^3+^/Co^2+^ redox couples and a surface enriched with lattice oxygen and electronically active oxygen species. These features collectively promote accelerated interfacial charge transfer and optimized hydrogen adsorption–desorption kinetics. As a result, the optimized CNO-HT interconnected nanosheet catalyst delivers a markedly reduced overpotential (86 mV), a smaller Tafel slope (103 mV dec^−1^), indicative of faster HER kinetics, and superior charge-transfer characteristics compared to CNO-U catalyst. Moreover, the catalyst maintains exceptional operational durability (96 h at 10 and 100 mA cm^−2^), sustaining stable hydrogen evolution for extended periods at both moderate and high current densities, with post-stability LSV and EIS analyses confirming the structural and electrochemical integrity of the catalyst. This work provides clear precursor-regulated nanosheet engineering, which is an effective route to unlock the intrinsic catalytic potential of spinel metal oxides. The insights gained herein offer a practical and scalable design principle for developing robust, cost-effective, and high-performance oxide electrocatalysts for sustainable hydrogen production.

## Data Availability

The original contributions presented in this study are included in the article/Supplementary Material. Further inquiries can be directed to the corresponding author.

## Supplementary Materials

The following supporting information can be downloaded at: https://www.mdpi.com/article/10.3390/ijms27031584/s1. References [51,52,53,54,55,56,57,58,59,60,61] are cited in Supplementary Materials.

## Abbreviations

The following abbreviations are used in this manuscript:

XRD	X-ray diffraction
FESEM	Field-emission scanning electron microscopy
XPS	X-ray photoelectron spectroscopy
EDAX	Energy-dispersive X-ray spectroscopy
Pt	Platinum
*C*_NFC_	Non-faradaic capacitance
*η*	Overpotential
*J*_NFC_	Non-faradaic capacitive current density
RHE	Reversible hydrogen electrode
SCE	Saturated calomel electrode
HER	Hydrogen evolution reaction
*ECSA*	electrochemically active surface area
EIS	Electrochemical impedance spectroscopy
LSV	Linear sweep voltammetry
CV	Cyclic voltammetry
*R*_ct_	Charge-transfer resistance
*N*	Number of moles of active catalyst
*F*	Faraday’s constant
TOF	Turnover frequency
PTFE	Polytetrafluoroethylene
*n*	Number of electrons transferred per hydrogen molecule
*A*	Geometric area of the electrode
