# Supplementary material for: Tuning the Hydrogen Evolution Activity of Co2NiO4 via Precursor-Controlled Synthesis
Source: Int J Mol Sci. 2026 Feb 5;27(3):1584. doi: 10.3390/ijms27031584 (PMC12897932; doi:10.3390/ijms27031584)
Supplement: Supplementary file 1 [file ijms-27-01584-s001.zip › ijms-4121019-supplementary.pdf]

Supporting Information

Tuning the Hydrogen Evolution Activity of Co_2NiO_4 via Precursor-Controlled Synthesis

Abu Talha Aqueel Ahmed [1], Momin M. Mujtaba [2], Kafeel Ahmed Tufail Ahmed [2], Abu Saad Ansari [3], Sangeun Cho [1], Youngmin Lee [1,4], Sejoon Lee [1,4] and Sankar Sekar [1,4,*]

[1] Division of System Semiconductor, Dongguk University, Seoul 04620, Republic of Korea; abutalha.aa@dongguk.edu (A.T.A.A.); ymlee@dongguk.edu (Y.L.); sejoon@dongguk.edu (S.L.)
[2] Department of Physics, M.S.G. Arts, Science & Commerce College, Malegaon-Camp, Malegaon 423203, India; mohammedmujtaba1318@gmail.com (M.M.M.); kafeelansari338@gmail.com (K.A.T.A.)
[3] Nano Center Indonesia Research Institute, Puspiptek Street, South Tangerang 15314, Banten, Indonesia; saad@nano.or.id
[4] Quantum-Functional Semiconductor Research Center, Dongguk University, Seoul 04620, Republic of Korea
* Correspondence: sanssekar@dongguk.edu

Table S1. Comparative electrocatalytic HER performance of the optimized Co_2NiO_4 catalyst and reported binary/ternary transition-metal oxide-based catalysts evaluated in 1.0 M KOH at 10 mA cm^{-2}.

No.	Catalyst film	Overpotential (mV) @10 (mA cm^{-2})	Tafel slope (mV dec^{-1})	Stability at *J* (*J* in mA cm^{-2})	Supporting Reference
1	$NiCo_2Se_4/NiCoS_4$	180	107.4	12 h@-1.3 V	[51]
2	$NiCo_2Se_4$	207	151.7	-	
3	P-doped $CuCo_2O_4$	152	115.8	15 h@-0.15 V	[52]
4	CuO@CuS	55	107	50 h@ 100	[53]
5	$CuCo_2O_4$	168	113	-	[54]
6	NCO@RuO_2-NCs	90	54.9	38 h@ 10	[55]
7	$CuCo_2O_4$	115	153	30 h@10	[56]
8	$NiCo_2O_4$/C2	468	91	25 h	[57]
9	$NiCo_2O_4$@C_3N_4	89	146	12 h@15	[58]
10	$MnCo_2S_4$	111	63	50 h@500	[59]
11	$MnCo_2O_4$	233	128	-	
12	Ru-$NiCo_2O_4$	25	43.4	45 h@10	[60]
13	$NiCo_2O_4$	167	88.1	-	
14	$NiCo_2S_4$@$NiCo_2O_4$-rGO	95	52	20 h@95 mV	[61]
**15**	**CNO-HT**	**127**	**103**	**100 h@10**<br>**100 h@100**	**Present work**

Table S2. Nyquist impedance fitted parameters value obtained using Z-view software for CNO-U and CNO-HT catalysts.

Catalysts	Before stability		After OER stability	
	R_S (Ω)	R_{ct} (Ω)	R_S (Ω)	R_{ct} (Ω)
**CNO-U**	0.459	8.307	-	-
**CNO-HT**	0.407	5.262	0.454	5.381

Supporting Figures

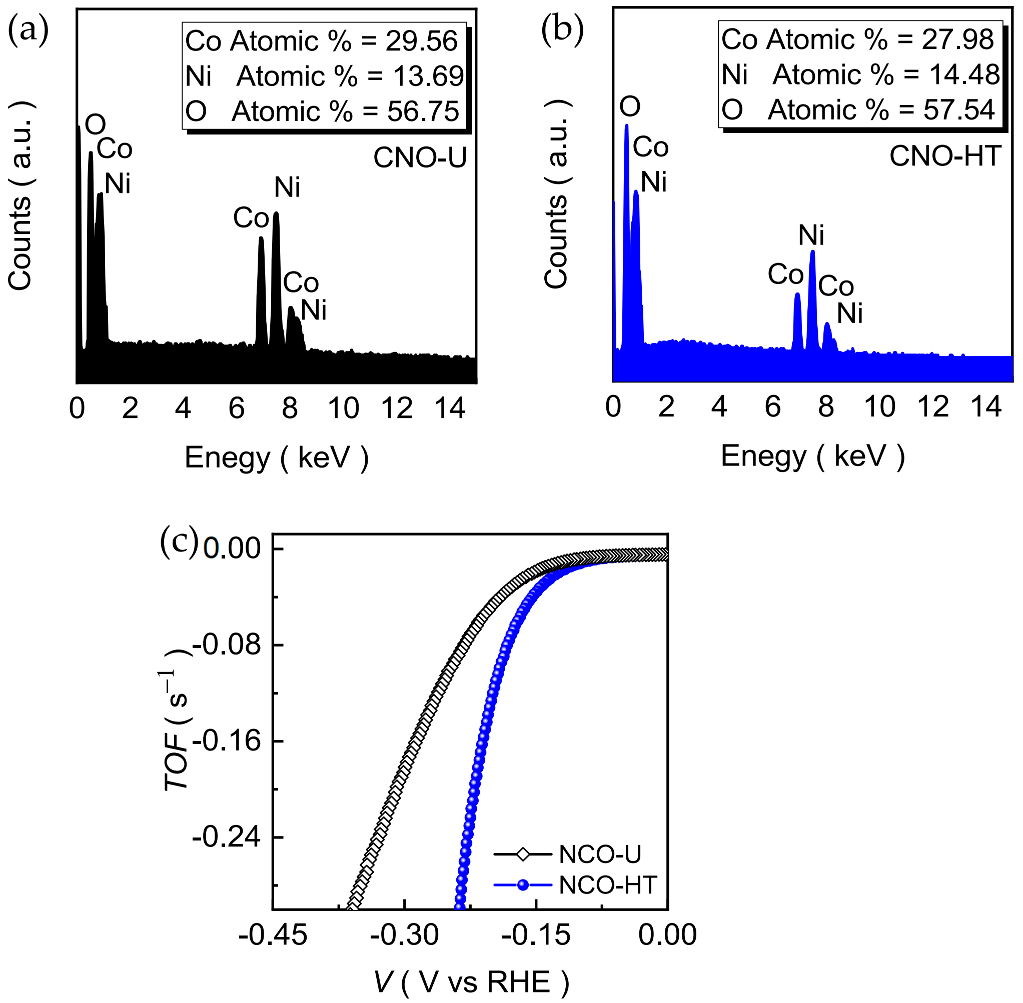

Figure S1. EDAX spectra of (**a**) CNO-U and (**b**) CNO-HT electrode films. The inset tables present the corresponding elemental compositions, highlighting the atomic percentage distribution of the detected elements. (**c**) TOF plots for CNO-U and CNO-HT electrode films.

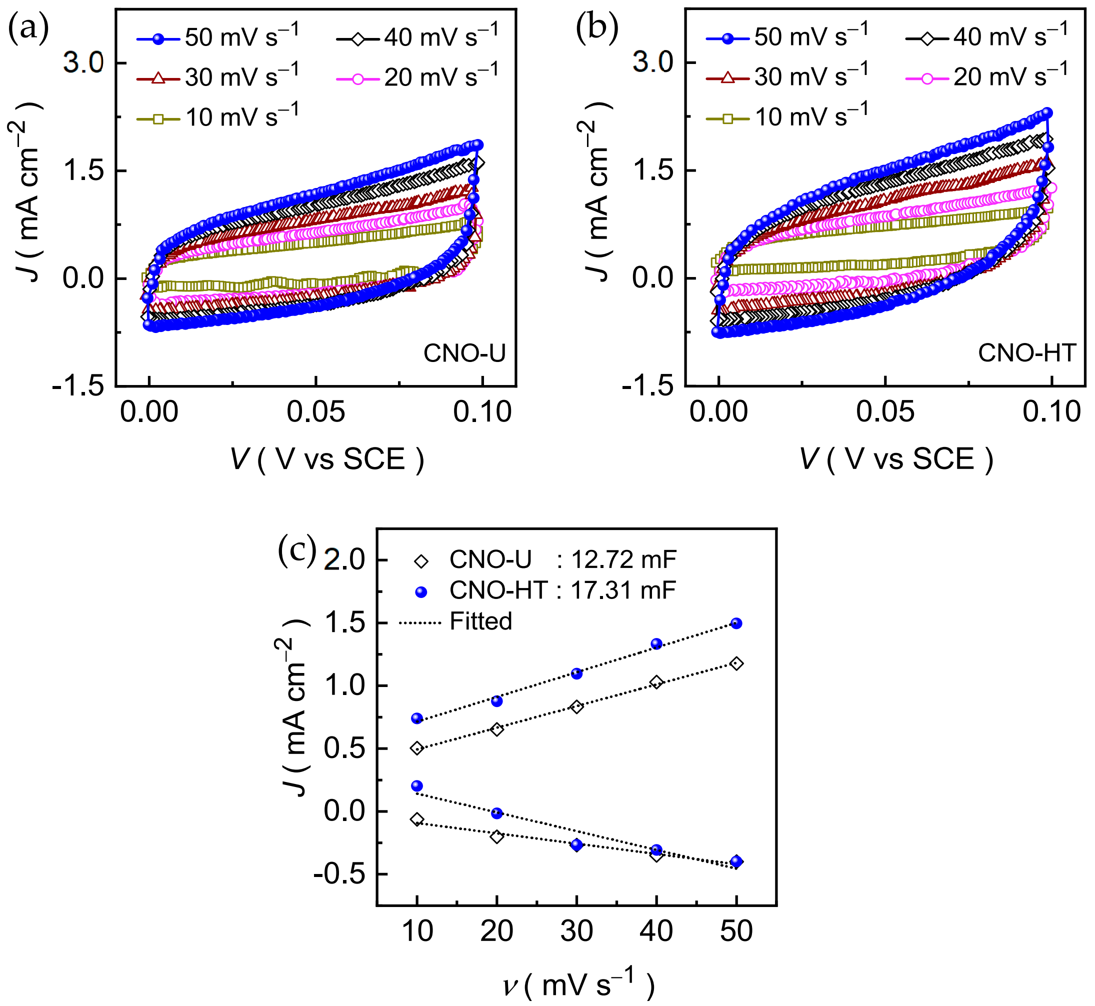

Figure S2. Non-Faradic CV curves recorded at various scan rates for (**a**) CNO-U and (**b**) CNO-HT catalysts. (**c**) Non-Faradaic current densities measured at 0.05 V (vs. SCE) across different scan rates were analyzed to evaluate the non-Faradaic capacitance (C_{NC}) and electrochemically active surface area (*ECSA*).

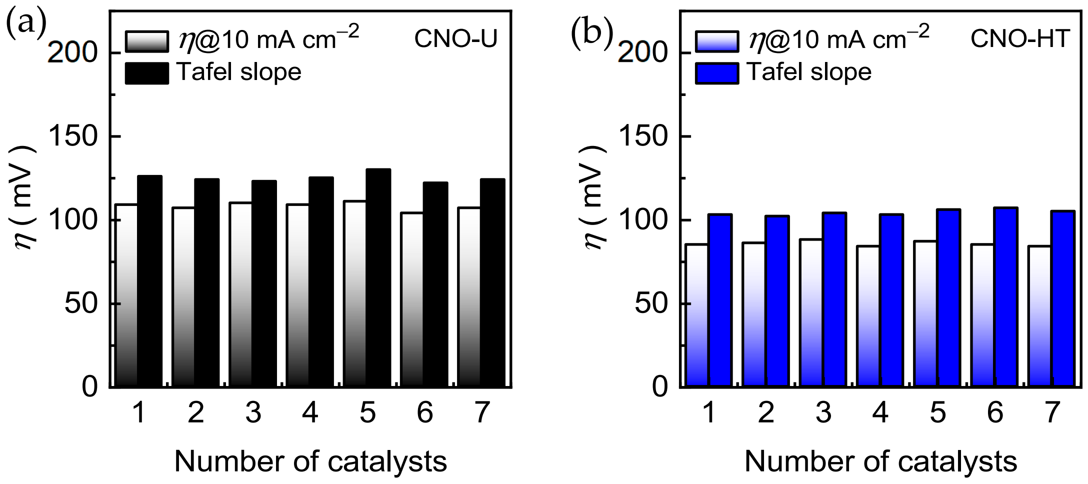

Figure S3. Reliability assessment of the (**a**) CNO-U and (**b**) CNO-HT catalysts evaluated using multiple independently prepared electrodes under identical experimental conditions.

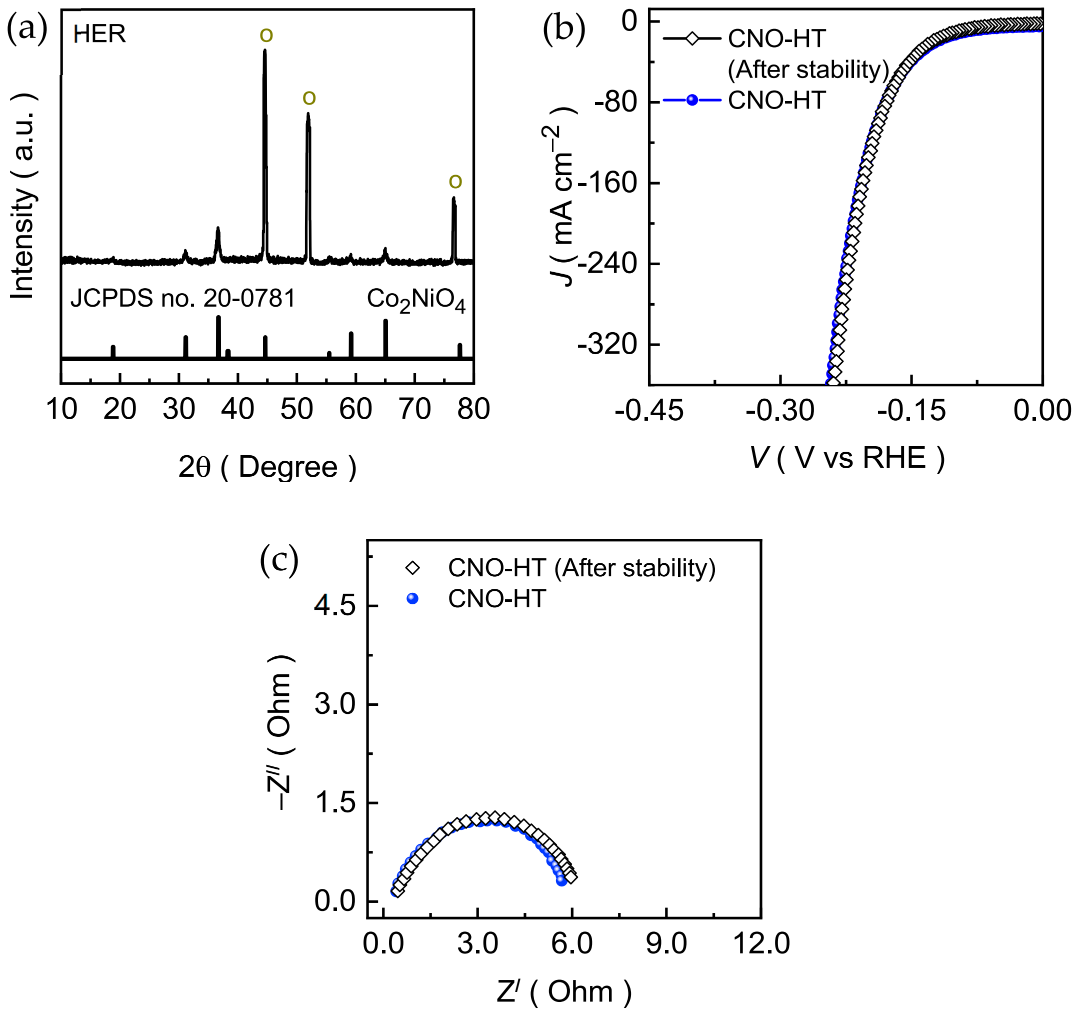

Figure S4. Post-stability recorded (**a**) XRD, (**b**) LSV, and (**c**) EIS curves of the CNO-HT catalyst measured under identical conditions to those of its pristine states in an alkaline electrolyte medium.
